# Feasibility, reliability, and validity of physical function tests and IADL survey questions in women living in rural, highland Ethiopia

**DOI:** 10.1371/journal.pone.0288828

**Published:** 2024-06-26

**Authors:** Jenna Golan, Anna Thalacker-Mercer, John Hoddinott

**Affiliations:** 1 Division of Nutritional Sciences, Cornell University, Ithaca, New York, United States of America; 2 Department of Cell, Development and Integrative Biology, University of Alabama at Birmingham, Birmingham, Alabama, United States of America; Universidad de Caldas, COLOMBIA

## Abstract

Physical function is the physical ability to fulfill one’s daily roles and responsibilities. Poor physical function is detrimental to health and income-generating activities. Unfortunately, there is a lack of validated methods to measure physical function in adult women in low- and middle-income countries, including Ethiopia, the locus of this study. This study evaluated the feasibility, reliability, and validity of physical tests, including the sit-to-stand (STS) and usual gait speed (UGS) and a context-appropriate instrumental activities of daily living (IADL) survey. The results of the STS were used to calculate a muscle quality index (MQI, STS accounting for body mass and leg length). Feasibility was ascertained qualitatively based on reports from the enumerators on their ability to administer the tests. Reliability was assessed by comparing the results of the tests and questions between each visit using either Cohen’s κ or Pearson’s ρ. The validity of MQI was assessed using relevant participant characteristics such as age and self-reported disability. The validity of the IADL was assessed using MQI. Study participants comprised 316 women between the ages of 18 and 45 years, living in rural Tigray, Ethiopia, who had previously participated in an impact evaluation of a safety net program. Over a one-week period, participants completed the STS and UGS tests and responded to the IADL survey questions three times. MQI was determined to be a feasible, reliable, and valid physical function test for women in rural, highland Ethiopia. UGS lacked feasibility and reliability; validity was not ascertained. The IADL questions were feasible and reliable, but validity was inconclusive. In rural Ethiopia, the MQI will be a valuable tool to develop interventions for improving physical function, which will have positive impacts on health and quality of life.

## Introduction

Physical function is a person’s ability to functionally, independently, and physically fulfill their activities of daily living (ADL), activities to take care of one’s own body, and instrumental activities of daily living (IADL), activities to support daily life within the home and community [1, 2]. It is both a cause and consequence of body size and composition and physical activity. Intrinsically, physical function is essential for overall health and quality of life [3, 4]. Instrumentally, physical function can identify people at risk for further declines in health [5–9], requiring additional care [4], or having future health expenditures [10]. Additionally, studies have used physical function as an outcome measure to determine the impact of interventions targeting health [11–13]. Outside of health measures, physical function impacts social networks [14, 15] and income-generating activities [16–18].

Medical practitioners and researchers in high-income countries have extensively measured physical function in a variety of populations, such as older adults with and without dementia [5, 19–23], people with chronic illness or pain (e.g., arthritis, asthma, and sarcopenia) [24–28], and obese people [22]. However, measuring physical function has been underused in low- and middle-income countries (LMIC) populations, with few exceptions [19, 29–31].

Measuring physical function in LMICs, such as Ethiopia, is essential because daily life is physically demanding. According to the 2016 Ethiopia Demographic and Health Survey (DHS), approximately 65% of the employed women living in rural Ethiopia were employed in skilled manual labor, unskilled manual labor, or agriculture, and 53% of women in rural households spent more than 30 minutes fetching drinking water daily [32]. Declines in physical function could significantly impact both necessary income-generating activities and activities needed for daily life.

Physical function is measured through physical tests or validated survey questions focused on the respondents’ ability to complete tasks required for personal care and independent living [33]. Several tests, including usual gait speed (UGS) and sit-to-stand tests (STS), do not require specialized training or resources, making them a viable option for measuring physical function in resource-limited settings [34]. UGS is considered a global marker of well-being [35]. The STS employs a motor pattern commonly used during IADLs [36]. Both tests collect continuous outcome measures, allowing data to be collected on a gradient at the upper end of the functional spectrum [5, 37, 38]. Prior studies have demonstrated the ability of UGS and STS to discriminate between functional levels in a young and active population [38].

Survey questions require less time and training to administer than physical tests, making them suitable for population studies. ADL and IADL surveys are validated survey questions used to measure physical function [39]. They use population-specific questions to determine how well participants complete their ADLs and IADLs [19, 40]. ADL surveys ask questions regarding bathing, dressing, and feeding oneself [1, 20, 33, 39]. IADL surveys ask about activities requiring more complex interactions, including housekeeping, grocery shopping, and managing finances [39, 41].

Physical function is not well understood in rural Ethiopia because the physical function assessment tools have not been studied in this setting. The objectives of this study were to 1) evaluate the feasibility of UGS, STS, and an IADL survey, 2) evaluate the reliability of UGS, STS, and an IADL survey, and 3) to evaluate the validity of UGS, STS, and an IADL survey to measure physical function in women living in rural highland Ethiopia. By understanding physical function in the region, future research could identify those needing additional healthcare, factors influencing health, and interventions to improve physical function.

## Materials and methods

### Study setting

Data were collected in the mountainous Central, Eastern, and South Zones of Tigray, Ethiopia. Most households in this region obtain their livelihoods from rainfed agriculture. The dry season occurs from January to June, and the main rainy season occurs between June and October. During the main rainy season, more than 90 percent of the country’s total crop production occurs, with harvesting occurring between October and December [42]. During the dry season, the poorest households in this region receive support from a government social safety net called the Productive Safety Net Program (PSNP). The PSNP provides cash or food payments for work on community projects such as terracing land, building roads, water reclamation, and reforestation [42, 43].

At the time of data collection, the villages where study households resided were remote. The average travel time to the market was 85 minutes on foot. Only 1% of households had water piped into their dwelling; the average time to fetch water and return was 45 minutes. Female respondents spent most of their time conducting childcare, cooking/ eating, domestic activities, and personal care during the dry season. During the main rainy season, they spent most of their time on childcare, cooking/eating, and food production activities [42]. Participants spent more time in sedentary/light exertion activities during the dry season than in the main rainy season [44] which may impact their physical function.

### Study subjects

Participants in this study resided in households that had previously taken part in an evaluation of the PSNP [42]. They were recruited based on the zones, counties, and villages where they lived. Within the zones, counties were excluded if the PSNP was inactive. Within each county, villages were excluded if they did not meet the criteria for a concurrently running study on physical exertion [44], which required two households that received benefits from the public works program and two that did not. All PSNP evaluation participants living in the selected villages were screened for eligibility. Prior studies that validated ADL and IADL surveys ranged in sample size from 30 [45] to over 350 people [46]. Inclusion criteria included women aged 18–45 years who were not pregnant and were not planning on leaving the area during data collection.

### Data collection

Data collection took place over two four-week periods. The first four-week period, the dry season round, took place April-May 2019 and coincided with the dry season when minimal agricultural activities occurred and the PSNP was operational. The second, four-week period, the agricultural round, took place September-October 2019 and occurred during the main rainy season when households were engaged in farming activities such as preparing the land, planting, and weeding. The PSNP was non-operational.

Over eight days, participants were visited up to six times. Participants answered survey questions during three visits, including the IADL questions, and completed the physical function tests. During the first visit, relevant anthropometric information, including body mass (Seca, Model 874 dr, Hamburg, Germany) and leg length, were measured. Women wore light clothing while being measured for body mass. Leg length was measured from the greater trochanter to the lateral malleolus using a standard measuring tape. All anthropometric measurements were measured twice, and the average of the two measurements was used for analysis. Additional anthropometric, demographic, and village information was collected during the PSNP evaluation [42].

### Physical function instruments

#### Sit-to-stand

A repeated STS test measures participants’ time to rise from a seated position without using their hands. There is no standardized protocol for this test, and several variations exist. For this study, we adapted the protocol used by Takai et al. [47] by modifying the seated position and the number of repetitions to make the test appropriate for this setting. The subjects started in a standing position, then sank into a low-seated squat before standing again. They squatted seven times as quickly as possible. The bottoms of their feet remained in contact with the ground throughout the test. Like other cultures, people in rural Ethiopia frequently sit in low-seated squats while resting or working near the ground (e.g., farming activities such as planting and weeding or domestic work such as cooking or laundry). Participants crossed their arms at the wrists and held them against their chest to ensure they were unaided in rising. A stopwatch recorded the time to the nearest 10^th^ of a second. The STS test was performed twice with an interval of 1 minute between the tests. The shorter of the two times was used for data analysis.

A muscle quality index (MQI), also referred to as the power sit-to-stand was calculated using Eq 1 [47–49].

$$\mathrm{MQI}=\ln\frac{L\times \mathrm{body}\;\mathrm{mass}\times g\times 7}{T_{\mathrm{sts}}}$$ (1)

Where: MQI is the muscle quality index, which estimates muscle power

L is the leg length measured from the greater trochanter to the lateral malleolus in centimeters

Body mass is measured in kg to the nearest tenth

g is the acceleration of gravity (9.8 m/s^2^)

T_sts_ is the time it took to complete the test

The MQI is a more complete measure than T_sts_ because it accounts for body mass and leg length, which modifies the relationship between T_sts_ and lower-extremity muscle strength [49]. This allows the MQI to better evaluate lower extremity function related to ambulation [48]. Further, it incorporates the velocity at which the muscle shortens (i.e., muscle power), which reflects the neuromuscular component [48]. Previous studies have found that the MQI, but not T_sts_, was correlated with muscle mass, measured by the cross-sectional area of the knee extensors, and muscle strength, measured by the maximum voluntary isometric knee extension force [47]. The MQI has been used to validate web-based physical activity interventions in community-dwelling older adults [50].

#### Usual gait speed

During the UGS, participants were timed as they walked 20 meters at their usual pace. Enumerators laid out pre-measured lengths of rope on a flat surface, free of obstacles, on stable ground. The participants started three steps behind the start of the pre-measured rope and continued walking until they were three steps beyond the rope. Time was recorded while the participants walked the 20-meter roped distance. The test was repeated three times, and the two closest times (out of three) were averaged and used for data analysis.

#### Instrumental activities of daily living survey

IADL surveys have been used to assess physical function since they were developed in 1963 [51]. Multiple versions of the survey exist. Each version asks the study participants if they experienced difficulty completing routine activities such as completing housework or grocery shopping. The routine activities selected for this version of an IADL survey were based on data from a "24-hour recall of time use" and "perceived energy exertion" survey module included in the evaluation of the PSNP. Three IADLs were selected that almost all participants had engaged in and were not expected to vary much in difficulty throughout the year. The IADLs survey questions collected information on participants’ perceived difficulty (i) traveling to and from the market (IADL—Market), (ii) cleaning and housework (IADL—Housework), and (iii) preparing food (IADL—Food) due to the physical nature of the activity.

The participants ranked the level of difficulty encountered on an ordinal scale: 1) experienced no difficulty, 2) experienced some difficulty, 3) needed assistance to complete the task, 4) avoided completing the task unless necessary, and 5) not responsible for the task. The responses to the IADL survey questions were collapsed into binary values determined by whether the participant experienced no difficulty or any level of difficulty. Participants who were not responsible for a task were marked as "missing." Additionally, a binary variable indicated whether a participant responded positively to experiencing difficulties with any IADLs (IADL–Any), and another variable captured the number of IADLs a participant reported difficulties (IADL–Number).

### Analysis

#### Feasibility of the tests

Feasibility was assessed based on the training and tools needed to administer the tests and qualitative reports from the enumerators on their ability to administer them. Characteristics that were considered included the availability of the tools locally, the space to conduct the tests, and the willingness of participants to complete the tests. During the agricultural round, participants were asked how they may alter their participation in the activities when they were unwell to determine the appropriateness of the activities included in the survey.

#### Reliability of the tests

The reliability of each physical function test was evaluated using the Pearson correlation coefficient (ρ). The physical function tests from each visit were compared to data from the other visits during that round. A coefficient between 0 to 0.20 was poor agreement, 0.21 to 0.40 was fair agreement, 0.41 to 0.60 was moderate/ acceptable agreement, 0.61 to 0.80 was substantial agreement, and 0.81 to 1.0 was near perfect agreement. These classifications have been used in other validation studies [52]. The reliability of each IADL question was evaluated using Cohen’s kappa statistic (κ). The same cutoffs were used to assess agreement for the correlation coefficient [53].

#### Validity of the tests

The validity of the MQI was assessed by evaluating the association of MQI with participant characteristics that are associated with physical function, including age, education, BMI, being underweight (BMI < 18.5 kg/m2), having a partner live in the household, being food insecure as measured by food gap [42], self-reported short- or long-term illness, and residing in a household that benefits from the PSNP. The validity of the UGS was not assessed because of the reliability analysis results.

The validity of the IADL survey questions was assessed with MQI due to the low agreement between UGS and MQI and the low agreement of UGS between visits. One visit per participant per round was randomly selected to analyze the validity of the IADL survey. First, a univariate analysis was conducted to determine if the responses to the IADL questions were significantly associated with MQI. Then, the variables were regressed with the rounds collapsed and by round. In rounds where the p-value for an IADL question was less than 0.20, the IADL question was fit into a multivariate regression with MQI as the dependent variable, and significant participant characteristics from that round were considered in the model. Participant characteristics considered for the multivariate model were the same as those used to assess the validity of physical function. The best-fit model was determined using backward-stepwise regression by Bayesian information criterion (BIC).

### Ethical approval

This study received ethical approval from the IRB at Cornell University (1902008596).

## Results

One hundred and seventy-eight women participated in the first round of data collection, the dry season round, and 138 women participated in the second round of data collection during the main rainy season, the agricultural round. One hundred and twenty-seven women participated in data collection during both rounds; 51 only participated during the dry season round and 11 during the agricultural round.

Between the two rounds of data collection, participants were comparable in age, education, BMI, marital status, PSNP beneficiary status, and their self-reported long-term and short-term illnesses and injuries, **Table 1**. The mean age of the participants was 30 years, and the range was 18 to 45. The average BMI was 19.4 ^kg^/_m_^2^. However, more participants were underweight in the dry season round than in the agricultural round (35.4% versus 31.6%). In addition, there was a seasonal difference in food security, with more participants reporting a food gap in the past six months during the dry season round (33.1%) than in the agricultural round (26.8%).

**Table 1:** Study participant characteristics.

Characteristic		Dry season round	Agricultural round	P-value
**N**		**178**	**138**	
Age (years)		30 (6.6)	30 (6.5)	0.46
Any education		50.0%	51.0%	0.90
BMI (^kg^/_m_^2^)		19.4 (2.1)	19.5 (2.3)	0.54
Underweight (BMI < 18.5 ^kg^/_m_^2^)		35.4%	31.6%	0.48
Partner lives in household		85.4%	83.3%	0.62
Food insecure during the past 6 months		33.1%	26.8%	0.23
Long-term illness or injury		15.7%	14.5%	0.76
Short-term illness or injury		24.7%	26.8%	0.67
Benefits from PSNP		47.2%	44.9%	0.69
MQI		9.9 (0.3)	9.9 (0.3)	0.89
UGS		8.7 (1.3)	8.5 (1.4)	0.26
IADL—Market		14.0%	21.0%	0.10
IADL—Housework		12.4%	20.3%	0.06
IADL—Food		12.4%	21.0%	0.04*
IADL—Any		17.4%	26.1%	0.06
IADL—Number				
	Zero	82.6%	73.9%	0.06
	One	5.1%	6.5%	0.58
	Two	3.4%	2.9%	0.81
	Three	9.0%	16.7%	0.04*

Values are presented as means with standard deviation in parentheses or as a percent of the study population. * Indicates that it is significant at α = 0.05.

The average values of the two physical function tests were similar between rounds. More participants reported difficulty with Any IADL and each IADL during the agricultural round compared to the dry season round. There was a statistically significant difference (p-value = 0.04) between those who reported difficulty preparing food for each round (12.4% vs 21.0%). During the dry season round, 82.6% of participants reported experiencing no difficulties with IADLs; this decreased to 73.9% in the agricultural round. In the dry season round, 9.0% of participants reported difficulties with all three IADLs. This increased to 16.7% of participants in the agricultural round (p-value = 0.04).

### Feasibility of the tests

The enumerators reported that the STS was feasible to administer to the study population and that there was space to administer the test in the participants’ households. Participants could complete the number of repetitions but reported that it was challenging, as they had started to fatigue. More difficulties were reported in administering the UGS. The UGS was not feasible to administer in some households because they did not have sufficient space that was flat, free of obstacles, and stable ground. It is an unsuitable test for use in rural Ethiopia. Participants reported that how they participated in the activities included in the IADL survey was likely to be altered when a participant was unwell.

### Reliability of the tests

For the reliability of the tests, the MQI had a near-perfect agreement for each visit within a round, with a correlation coefficient greater than 0.80 (**Table 2**), except for visit 1 compared to visit 3 in the dry season round when there was substantial agreement. Overall, UGS had a lower agreement between visits within rounds, with most comparisons having moderate agreement. There was substantial agreement between visits 2 and 3 in the agricultural round (correlation coefficient = 0.62). There was nearly always poor agreement between the UGS and MQI between each visit for both rounds.

**Table 2:** Reliability of physical function tests.

		Dry season round		Agricultural round	
Test	Visit numbers	N	Pearson’s ρ	N	Pearson’s ρ
MQI					
	1 vs 2	176	0.81****	126	0.88****
	1 vs 3	147	0.78***	122	0.82****
	2 vs 3	147	0.81****	129	0.84****
UGS					
	1 vs 2	176	0.54**	124	0.59**
	1 vs 3	151	0.40*	117	0.40*
	2 vs 3	152	0.47**	121	0.62***
MQI vs UGS					
	Visit 1	175	-0.22*	127	-0.12
	Visit 2	176	-0.10	132	-0.05
	Visit 3	147	-0.18	124	-0.05

* Indicates fair agreement

** indicates moderate/ acceptable agreement

*** indicates substantial agreement

**** indicated near-perfect agreement.

The responses to each IADL question were compared between visits in each round, **Table 3.** During the dry season round, there was a near-perfect agreement in the responses to the IADL questions between the visits (κ ranged from 0.78 to 0.97). During the agricultural round, there was either substantial or near-perfect agreement (κ ranged from 0.70 to 0.88).

**Table 3:** Correlation of IADL questions.

		Dry season round		Agricultural round	
IADL question	Visit numbers	N	Cohen’s κ	N	Cohen’s κ
IADL—Market					
	1 vs 2	174	0.89****	128	0.81****
	1 vs 3	150	0.81****	124	0.83****
	2 vs 3	150	0.89****	131	0.80***
IADL—Housework					
	1 vs 2	175	0.97****	128	0.77***
	1 vs 3	149	0.85****	124	0.70***
	2 vs 3	149	0.88****	131	0.84****
IADL—Food					
	1 vs 2	176	0.97****	128	0.84****
	1 vs 3	150	0.85****	124	0.86****
	2 vs 3	150	0.88****	131	0.88****
IADL—Any					
	1 vs 2	176	0.91****	128	0.76***
	1 vs 3	151	0.78***	124	0.70***
	2 vs 3	151	0.84****	131	0.71***
IADL—Number					
	1 vs 2	177	0.91****	137	0.74***
	1 vs 3	177	0.75***	137	0.65***
	2 vs 3	177	0.79***	137	0.71***

* Indicates fair agreement

** indicates moderate/ acceptable agreement

*** indicates substantial agreement

**** indicated near-perfect agreement.

### Validity of the tests

**Table 4** shows the association between MQI and participant characteristics and IADL responses with the rounds combined and by round. Age, BMI, underweight, and self-reported long-term illness or injury were consistently significantly associated with MQI_._ (p-value < 0.05) demonstrating it to be a valid measure of physical function. For every year’s increase in age, physical function decreased as measured by the MQI. An increase in BMI was associated with improvements in physical function as measured by MQI, while those underweight had lower physical function as measured by MQI. Those who self-reported a long-term illness or injury had a lower physical function than those who did not.

**Table 4:** Association of MQI with participant characteristics and IADL responses.

	All		Dry season round		Agricultural round	
Model parameter	Coefficient	p-value	Coefficient	p-value	Coefficient	p-value
N	316		178		138	
Age	-0.01	0.01**	-0.01	0.06*	-0.01	0.04**
Any education	-0.02	0.46	-0.01	0.88	-0.04	0.30
BMI (^kg^/_m_^2^)	0.04	<0.01***	0.05	<0.01***	0.03	<0.01***
Underweight (BMI < 18.5 ^kg^/_m_^2^)	-0.15	<0.01***	-0.18	<0.01***	-0.11	0.02**
Partner lives in household	0.05	0.29	0.05	0.41	0.04	0.50
Food insecure	-0.05	0.13*	-0.01	0.78	-0.11	0.02**
Long term illness	-0.13	0.04**	-0.13	0.04**	-0.14	0.02**
Short term illness	0.01	0.78	0.02	0.74	-0.04	0.37
Safety net beneficiary	0.04	0.17*	0.03	0.51	0.06	0.16*
IADL–Market	-0.01	0.82	0.08	0.21	-0.10	0.07*
IADL–Housework	0.02	0.59	0.12	0.09*	-0.06	0.29
IADL–Food	0.03	0.52	0.12	0.08*	-0.05	0.33
IADL—Any	0.03	0.53	0.12	0.08*	-0.05	0.33
Number of IADLs	0.01	0.74	0.04	0.09*	-0.03	0.17

The mean value of ln(MQI) for each round was 9.92 with a standard deviation of 0.30 for round 1 and 0.25 for round 2.

* Indicates statistical significance less than 0.20, qualifying for consideration in the multivariate model

** Indicates statistical significance at <0.05

*** Indicates statistical significance <0.01

Among the IADL responses, none were significantly associated with MQI when the rounds were combined. During the dry season round, IADL–Housework, IADL–Food, IADL–Any, and IADL–Number were significant at the α = 0.10. Only the IADL—Market was significant at the α = 0.10 level during the agricultural round.

Multivariate models were constructed for each IADL question where the response was significant at α = 0.10, **Table 5**. In the dry season round, the IADL—Housework and IADL—Any were significant after controlling for BMI. The IADL—Food and IADL–Number were significant after controlling for BMI and self-reported long-term illness or injury. For the agricultural round, IADL-Market was significant after controlling for BMI. In the dry season round, all significant IADL variables had a positive coefficient, indicating that they were positively associated with MQI and had improved physical function compared to those who responded that they had no difficulties. During the agricultural round, IADL—Market had a negative coefficient, indicating that those who reported difficulty had lower MQI and lowered physical function than those who responded that they had no difficulties.

**Table 5:** Multivariate model IADLs and MQI.

		IADL		BMI		Long-term illness or injury	
IADL question		Coefficient	p-value	Coefficient	p-value	Coefficient	p-value
Dry season round							
	Housework	0.13	0.04**	0.05	0.02**		
	Food	0.15	0.02**	0.05	<0.01***	-0.12	0.04**
	IADL—Any	0.12	0.03**	0.05	<0.01***		
	IADL—Number	0.05	0.03**	0.05	<0.01***	-0.12	0.05**
Agricultural round							
	Market	-0.10	0.04**	0.03	<0.01***		

MQI is the dependent variable in the model. The mean value of MQI for each round was 9.92 with a standard deviation of 0.30 for round 1 and 0.25 for round 2.

* Indicates statistical significance less than 0.20

** Indicates statistical significance at <0.05

*** Indicates statistical significance <0.01

## Discussion

This research found that MQI is a feasible, reliable, and valid method to measure physical function in adult women living in rural highland Ethiopia. Measuring MQI required very little training or materials, making it a low-cost, feasible option for research conducted in resource-constrained settings. Additionally, enumerators reported that the test was easy to administer and acceptable to the study participants. MQI had high reliability with a near-perfect agreement between visits in each round. Additionally, MQI was associated with age, BMI, and self-reported long-term illness or injury, indicating it is a valid measure of physical function. MQI is a promising tool for measuring physical function in similar populations.

UGS was neither feasible nor reliable, and it is not likely a good measure of physical function in adult women living in rural highland Ethiopia. UGS was not feasible because many homes did not have sufficient space that was free of obstacles, flat, and stable. The test was frequently administered in the nearest space that was suitable, but this likely contributed to its lack of reliability. The space where the test was administered may have been rocky or in a public area, which could have resulted in the participants varying their gait speed.

The IADL survey was reliable, but it had inconclusive validity. The high reliability of the subjective scoring for each IADL among the rounds indicates that the participants understood the question and interpreted it consistently. There are several potential reasons that they survey lacked validity. The sample size may have been too small especially since the majority of participants reported having no difficulties completing the activities (82.6% during the dry season round and 73.9% during the agricultural round). Additionally, changes in physical function may not be reflected in the IADL responses because no one was able to fulfill these responsibilities in place of the study participant. Finally, people may have experienced difficulties in completing these activities that were not related to physical function. In older populations, social participation and mental health were associated with IADL scores [54, 55]. More work is needed to develop an IADL survey for this population in rural Ethiopia. Additional research should be conducted to understand why women reported difficulties in their IADL that were not associated with their MQI.

The strengths of this research include the careful attention given to the development of the methodologies and the administration of the STS and UGS in a novel setting. Using MQI as a measure of physical function is a strength of this paper. To the authors’ knowledge, MQI has not previously been used in rural regions of LMIC. Previous work on physical function in LMICs used grip strength [29, 31, 56]. MQI is a better measure of physical function than grip strength because it requires muscle strength, mobility, balance, and coordination [57]. In addition, MQI is more likely to reflect the necessary activities to fulfill a study participant’s daily roles and responsibilities [36].

There were limitations in this paper, particularly around the development of the IADLs. The three activities included in the IADL survey were selected because they appeared in most responses in the time-use module of the PSNP evaluation. Additionally, there was minimal seasonal variation in the frequency or difficulty of completing these activities. Unfortunately, logistical and time constraints prevented the piloting of the IADL survey. Respondents reported that their participation in the activities included in the IADL survey would change when they were unwell, indicating that the activities should be an appropriate measure of physical function. However, this relationship was not reflected in this study’s data. There is a lack of information to help us understand why the IADL survey questions had limited validity or to aid in creating a new survey. More research is needed to explore survey questions and activities that accurately characterize this population’s physical function. While the IADL survey may not detect a change in physical function, participants reported difficulties completing the activities. These responses should not be ignored or dismissed; research is needed to understand the cause of these reported difficulties.

Future research that includes MQI should examine several additional factors that impact physical function. This research’s nutritional status was limited to BMI, but it should include body composition. Physical function is both a cause and a consequence of lean body mass, making it an important consideration when evaluating physical function. Additionally, more detailed dietary intake and nutritional biomarkers should be measured. Diet quality, especially protein and micronutrient intake, impacts physical function [58–60]. Physical activity, including type, intensity, and duration, should be included in future research. In this study, medical history was limited to self-reported long- or short-term illness or injury. However, an in-depth medical history would be valuable to future research, including the type, timing, treatment, and health-seeking behaviors around illnesses and injuries. Finally, research regarding physical function needs more frequent data points over longer time periods. There are seasonal variations in many factors that influence physical function, including diet [61], physical activity [42], and exposure to diseases [62]. More data points will help determine how quickly physical function changes in women in rural Ethiopia in response to different factors and if there are long-term changes.

## Conclusions

Measuring physical function and identifying those experiencing difficulties completing their IADLs is essential in all vulnerable populations, including women in rural Ethiopia. IADLs in rural highland Ethiopia are physically demanding. Taking care of oneself, family, and household, as well as many income-generating activities, require significant manual labor and traveling significant distances on foot. Therefore, decreases in physical function could affect the well-being of one’s family by impacting household income, diet quality, and health through water, sanitation, and hygiene.

A feasible, reliable, and valid measure of physical function is vital in addressing poverty and malnutrition. It would allow healthcare providers to identify those requiring additional medical care and researchers to identify factors influencing physical function, including nutritional, behavioral, and environmental factors, and develop potential interventions to improve physical function. This research has demonstrated that the MQI, using a timed seven-repetition stand to seated squat, is a feasible, reliable, and valid measure of physical function in this setting.
